# Sheathless Microflow Cytometry Using Viscoelastic Fluids

**DOI:** 10.1038/s41598-017-12558-2

**Published:** 2017-09-27

**Authors:** Mohammad Asghari, Murat Serhatlioglu, Bülend Ortaç, Mehmet E Solmaz, Caglar Elbuken

**Affiliations:** 1 UNAM - National Nanotechnology Research Center, Institute of Materials Science and Nanotechnology, Bilkent University, 06800 Ankara, Turkey; 2 Department of Electrical and Electronics Engineering, Izmir Katip Celebi University, 35620 Izmir, Turkey

## Abstract

Microflow cytometry is a powerful technique for characterization of particles suspended in a solution. In this work, we present a microflow cytometer based on viscoelastic focusing. 3D single-line focusing of microparticles was achieved in a straight capillary using viscoelastic focusing which alleviated the need for sheath flow or any other actuation mechanism. Optical detection was performed by fiber coupled light source and photodetectors. Using this system, we present the detection of microparticles suspended in three different viscoelastic solutions. The rheological properties of the solutions were measured and used to assess the focusing performance both analytically and numerically. The results were verified experimentally, and it has been shown that polyethlyene oxide (PEO) and hyaluronic acid (HA) based sheathless microflow cytometer demonstrates similar performance to state-of-the art flow cytometers. The sheathless microflow cytometer was shown to present 780 particles/s throughput and 5.8% CV for the forward scatter signal for HA-based focusing. The presented system is composed of a single capillary to accommodate the fluid and optical fibers to couple the light to the fluid of interest. Thanks to its simplicity, the system has the potential to widen the applicability of microflow cytometers.

## Introduction

Flow cytometry is a well-established technique for automated multi-parameter analysis of suspended cells and particles for biomedical applications and clinical research. Immunophenotyping using flow cytometry is one of the most commonly performed assays in clinics^1^. Commercially available benchtop flow cytometers mostly use optical detection that requires the interaction of a laser beam with the sequentially aligned particles of interest in a flow cell.

Optical flow cytometer has been around since 1960s^2^, and yet traditional flow cytometers are bulky, expensive and require technical labor for maintenance and operation. Fortunately, microfluidics has addressed these challenges, which gave birth to microfluidic-based flow cytometers. The developed microflow cytometers mostly focus on advancements in flow cell design by integrating the optical components and miniaturization, thus lowering the required sample volume and cost while achieving portability.

Various strategies have been used for flow cytometers to achieve better optical interaction of the laser beam with the particles of interest. Aligned optical fibers^3–8^, inscribed optical waveguides^9–11^, and microfabricated customized lenses^12–16^ were used in optical interrogation region to obtain better light interaction with focused particles without using any free-space optics. Inscribing waveguides is a complex fabrication process, and brings further challenges such as alignment issues during the coupling of fiber optics, laser and detector sources. Instead, direct assembly of already coupled optical fibers provides mechanical stability, ease of alignment and efficient light interaction with individual particles on an integrated chip.

Additionally, several microfluidic techniques have been developed to achieve 3D focusing of particles into the optical interrogation region to obtain higher repeatability and lower coefficient of variation (CV) values. Sheath flow supported hydrodynamic focusing^5,10,17–19^ requires squeezing of the suspension fluid with sheath fluid in order to create a narrow, size-adjustable width of sample flow in microfluidic channel. Such systems suffer from the use of extensive amount of sheath fluid, require precise fabrication, and high accuracy on flow control. In addition to hydrodynamic focusing, electric^20,21^, magnetic^22^ or acoustic forces^23,24^ can be used to focus the particles along the same line, which we refer as 3D focusing (in some studies, called as 2D focusing due to focusing forces along both lateral dimensions^25,26^). These methods require additional actuation forces to induce particle focusing and increase the fabrication and operation complexity^27^.

On the other hand, inertial^28–31^ and viscoelastic focusing^32–38^ techniques allow sheathless single inlet/outlet chips, which provide simplicity in fabrication and operation. Both techniques are highly dependent on fluid properties and flow regime. For Newtonian fluids, inertial lift force causes the particles to migrate across the velocity streamlines due to velocity gradient across channel cross section. When inertial forces are dominant compared to viscous forces, particles move to equilibrium positions inside medium. Inertial focusing has been investigated for straight channels of different geometries including circular^39^, square^40^, rectangular^41^ and even unusual cross sections^42^, resulting in multiple-line focusing. Implementing curved structures creates Dean drag force which results in focusing of particles into a narrower region^29,43^.To reach single-train of particles, the structure of the channel should be engineered or different structures of channels should be integrated in a specific sequence.

Recently, non-Newtonian viscoelastic fluids have attracted great attention^44–49^. Inertial focusing is implemented by optimizing the channel dimensions, channel geometry, and flow rate for a specific particle size. Viscoelastic fluids provide an additional degree of freedom which is the ability to tune the rheological properties of the carrier fluid. This results in single-line focusing of particles in simple geometries. Viscoelastic fluids are prepared by adding biological^50,51^ or synthetic polymeric powders^52^ to Newtonian solvents. These fluids show viscoelastic behavior and generate elastic foce that affects the equilibrium position of suspended particles.

**Figure 1:**
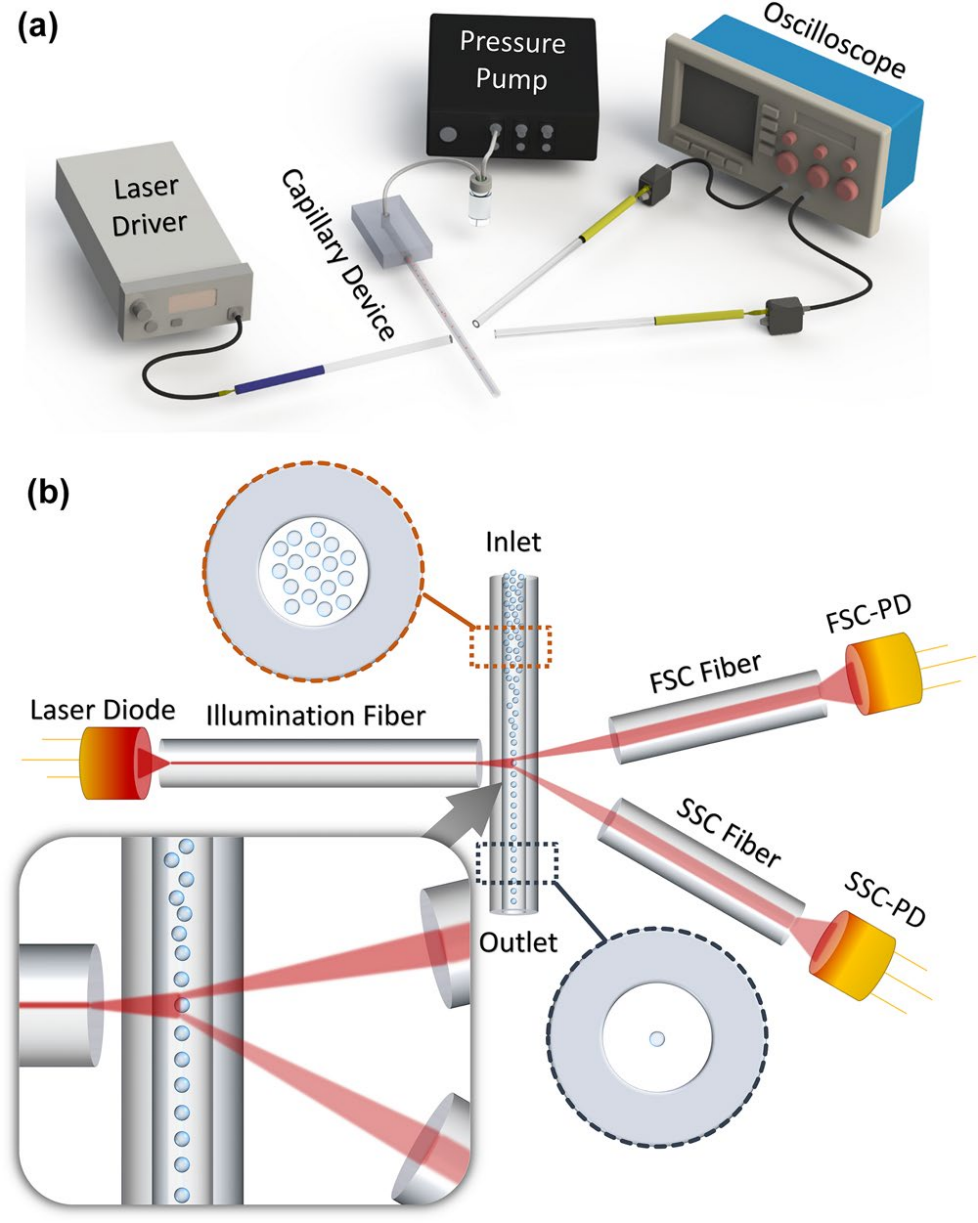
(**a**) Schematic illustration of sheathless microflow cytometer setup consisting of the assembled chip, a 635 nm laser diode, a laser driver, a pressure pump, an oscilloscope, and Si photodetectors. (**b**) Schematic illustration of assembled components (not to scale). Particles are focused at the center of circular capillary channel due to viscoelastic forces. (inset) When individual particles arrive at the laser interaction region, they scatter laser light (forward scatter, FSC and side scatter, SSC) collected with photodetector (PD) coupled multimode fibers. PD signal was acquired with oscilloscope and analyzed in MATLAB to get flow cytometry parameters.

In this paper, we combine fiber-based microflow cytometer with viscoelastic particle focusing achieving a new sheathless microflow cytometer as schematically shown in Fig. 1 (for details, see Materials and Methods section). To the best of our knowledge, this is the first study presenting viscoelastic focusing in a microflow cytometer. Three non-Newtonian fluids have been evaluated for achieving high performance 3D particle focusing. Then, the optical detection system was developed by assembling a 15 cm-long capillary as the flow channel and three optical fibers to incorporate the laser light and to collect forward scatter (FSC) and side scatter (SSC) signals. The system does not require any microfabrication steps and can be assembled on a finger-sized substrate. Finally, our results show that hyaluronic acid (HA) and polyethylene oxide (PEO) with specific molecular weight can be used to focus 6 µm diameter particles to the center of the capillary at high accuracy, yielding a cytometer throughput of over 750 events/s and coefficient of variation (CV) of 5.8%.

## Theory

Elastic behavior of viscoelastic fluid originates from the suspension of polymers inside solution. This non-Newtonian behavior induces elastic force due to normal stress differences^53^. Under non-Newtonian Poiseuille pipe-flow, two normal stress differences can be defined as

1 $${N}_{1}={\sigma }_{zz}-{\sigma }_{rr}$$

2 $${N}_{2}={\sigma }_{rr}-{\sigma }_{\theta \theta }$$

where *z*, *r*, and *θ* denote flow, radial, and vorticity directions, respectively. Diluted, constant shear viscosity solutions are considered to be Boger fluids^54^ and can be studied by Oldroyd-B model. For Boger fluids, the second normal stress difference (${N}_{2}$) is negligible compared to the first normal stress difference (${N}_{1}$). Therefore, elastic force (*F*_*e*_) can be written as^55^

3 $${F}_{e}\approx {a}^{3}\frac{\partial {N}_{1}}{\partial r}$$

To characterize elasticity, Weissenberg number is defined as

4 $$Wi=\lambda \dot{\gamma }$$

where $\lambda$ is relaxation time and $\dot{\gamma }$ is shear rate. To compare elasticity and inertia, elasticity number is defined as

5 $$El=\frac{Wi}{\mathrm{Re}}$$

In pipe flow, radial migration velocity for a particle under the effect of elastic force is given as^56^

6 $${V}_{r}\propto {a}^{2}Wi\frac{\partial \dot{\gamma }}{\partial r}$$

To relate the radial migration velocity to geometrical parameters and solution properties, equation (6) can be written as^57^

7 $${V}_{r}\propto \frac{0.77\frac{c}{{c}^{\ast }}}{1+0.77\frac{c}{{c}^{\ast }}}Wi{(\frac{a}{R})}^{2}\frac{r}{R}$$

where ${c}$, ${c}^{\ast }$, $a$, $R$ and $r$ are polymeric concentration, overlapping polymer concentration, particle radius, channel radius and radial position of particle, respectively.

## Results and Discussion

### Numerical results

**Figure 2:**
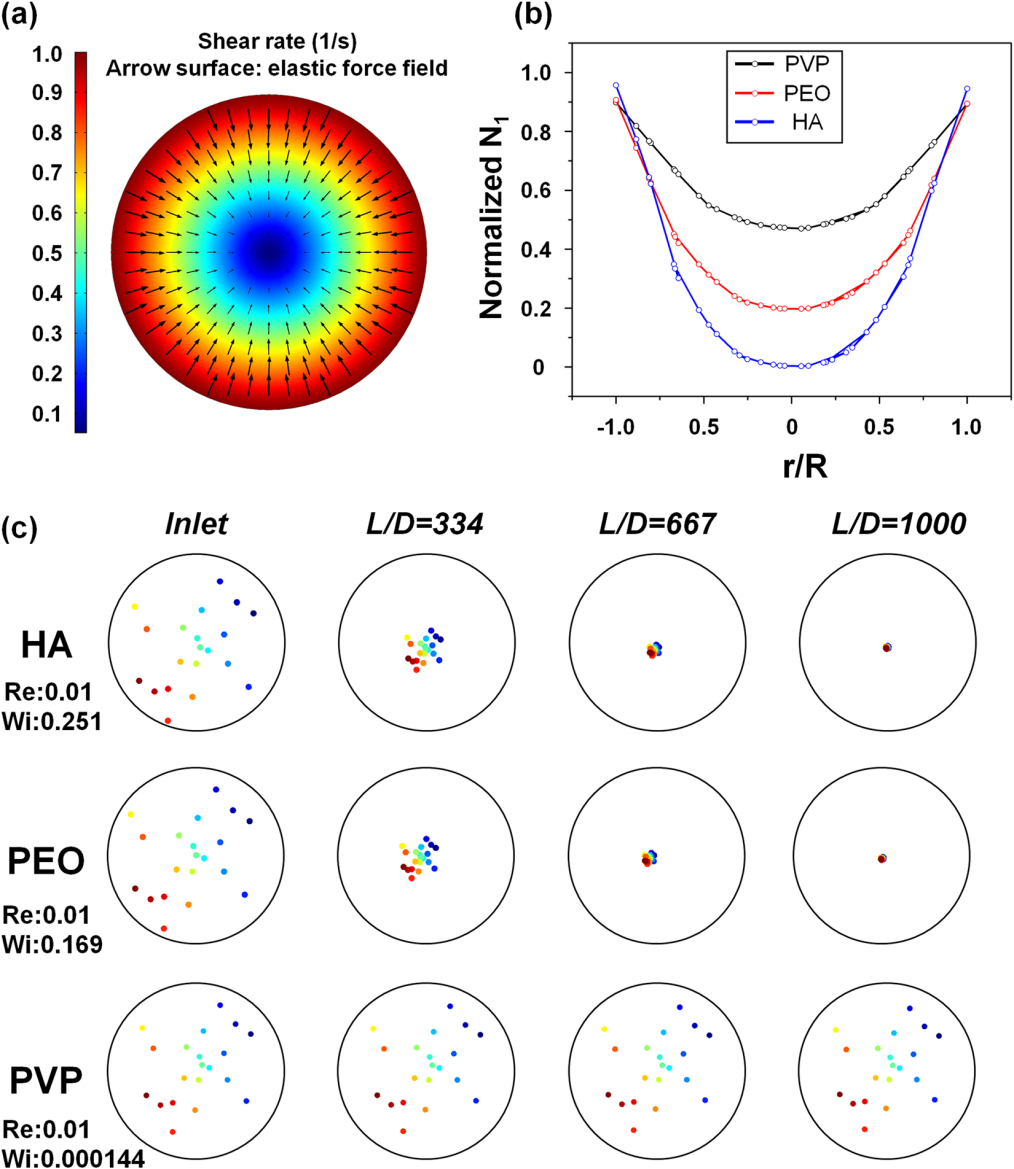
Numerical results of viscoelastic focusing: (**a**) Normalized shear rate for viscoelastic fluid inside cylindrical capillary tube and corresponding elastic force field. (**b**) Normalized first normal stress difference versus dimensionless radial position, *r/R* for PVP, PEO, and HA. (**c**) Cross-sectional position of twenty particles along the capillary microchannel, with 2 cm intervals from the inlet. Here, L and D represent the axial distance from inlet and channel diameter, respectively.

Elastic force acting on suspended particles in the medium is dependent on particle size and first normal stress difference gradient (equation (3)). First, we studied the focusing performance of three viscoelastic solutions using numerical simulations. Lateral velocity depends on shear rate gradient as shown in equation (6). Under viscoelastic Poiseuille pipe-flow, shear rate decreases from wall to center of the pipe as shown in Fig. 2a. In our study, we tested 6 µm diameter particles suspended in three different viscoelastic media. ${N}_{1}$ was numerically calculated for these fluids, and the results are given in Fig. 2b. HA has the highest ${N}_{1}$ gradient whereas PVP has the lowest value. This result suggests that particles in HA solution can be focused faster due to higher elastic force.

Radial migration of particles was analyzed using particle tracing module. Elastic force (equation (3)) and drag force *F*_*d*_ = 6*πμau* were defined using ${N}_{1}$ and relative velocity (*u*), which were obtained from laminar flow simulation. Neutral buoyancy was assumed by taking the density of the solutions and the particles the same (ρ = 1050 kg/m^3^). Particle-particle interactions were not considered. Twenty particles suspended in the medium were traced during the simulations.

Figure 2c shows cross-sectional position of particles at increasing distances from the inlet (L: axial distance from inlet, D: channel diameter). Particles suspended in PEO and HA solutions were focused at the centerline while PVP showed dramatically lower performance.

### Analytical results

As it can be observed from our numerical results, viscoelastic focusing is highly dependent on the rheological properties of the fluids. A similar analysis can be made based on the analytical model given in the previous section. According to equation (7), particle migration velocity (*V*_*r*_) is a function of polymeric concentration (*c*), overlapping polymer concentration (c*), and relaxation time (*λ*) for a given combination of shear rate ($\dot{\gamma }$), blockage ratio (*a/R*) and radial position (*r/R*). A rheological term can be defined as $\frac{0.77\frac{c}{{c}^{\ast }}}{1+0.77\frac{c}{{c}^{\ast }}}\lambda$ that reflects the influence of the viscoelastic fluid on particle migration velocity. Relaxation time for polymers was estimated using the Zimm relaxation time $\lambda  \sim 4\pi {\eta }_{s}{R}_{g}^{3}/{k}_{B}T$ where ${k}_{B}$,*T*, ${\eta }_{s}$, and ${R}_{g}$ correspond to Boltzmann constant, temperature, solvent viscosity, and radius of gyration of polymer^58^, respectively. ${R}_{g}$ for ${{PEO}}_{5{MDa}}$ ^59^, ${{HA}}_{1.06{MDa}}$ ^60^, ${{PVP}}_{40{kDa}}$ ^61^ were found from the literature. ${c}^{\ast }$ values were determined by using Graessley’s modified equation^62^ where ${c}^{\ast }=0.77/[\eta ]$. $[\eta ]$ is the intrinsic viscosity which  depends on polymer type and its molecular weight. ${[\eta ]}_{\mathrm{PEO}}$, ${[\eta ]}_{\mathrm{PVP}}$, and ${[\eta ]}_{\mathrm{HA}}$ were found from the literature^60,63,64^ as7.2 × 10^−2^ (*M*_*w*_)^0.65^ ≈ 1628 *ml* $/g$, 3.93 × 10^−2^(*M*_*w*_)^0.59^ ≈ 20.4 *ml/g* and 3.21 × 10^−2^(*M*_*w*_)^0.783^ ≈ 1676 *ml/g*, respectively. The calculation of the rheological term is given in Table 1 for PEO, PVP, and HA. As seen from this analysis, PEO and HA yielded higher $\frac{0.77\frac{c}{{c}^{\ast }}}{1+0.77\frac{c}{{c}^{\ast }}}\lambda$ and similar values, thus leading to faster focusing of the particles. These results are in good agreement with the numerical simulation results.

**Table 1:** Calculation of the rheological term used for particle velocity analysis.

Fluid	${{\boldsymbol{R}}}_{{\boldsymbol{g}}}$ (nm)	${\boldsymbol{c}}$ (g/ml)	$[{\boldsymbol{\eta }}]$ (ml/g)	${{\boldsymbol{c}}}^{{\boldsymbol{\ast }}}$ (g/ml)	${\boldsymbol{\lambda }}$ (ms)	$\frac{{\bf{0.77}}\frac{{\bf{c}}}{{{\bf{c}}}^{{\boldsymbol{\ast }}}}}{{\bf{1}}+{\bf{0.77}}\frac{{\bf{c}}}{{c}^{{\boldsymbol{\ast }}}}}{\boldsymbol{\lambda }}$(ms)
PEO	155.4	0.0005	1628	0.000473	11.39	5.11
PVP	14.4	0.08	20.40	0.037747	9.06 × 10^−3^	5.62 × 10^−3^
HA	126.1	0.005	1676	0.000459	6.08	5.43

### Experimental results on particle focusing

The numerical and analytical results demonstrate the effect of the rheological properties of the fluids on the focusing performance. In this section, we analyze the experimental particle focusing performance for all three viscoelastic fluids using the setup explained in the Materials and Methods section. The inlet pressure was adjusted so that $\mathrm{Re} < 1$ and therefore inertial effect was neglected. To analyze the performance of focusing, we calculate the probability distribution function (PDF) for each measurement. The observations were made using the high-speed camera and calculations were performed from the movies recorded at a distance of 6 cm from the capillary inlet. A short movie clip showing our particle focusing results is provided as supplementary video file.

**Figure 3:**
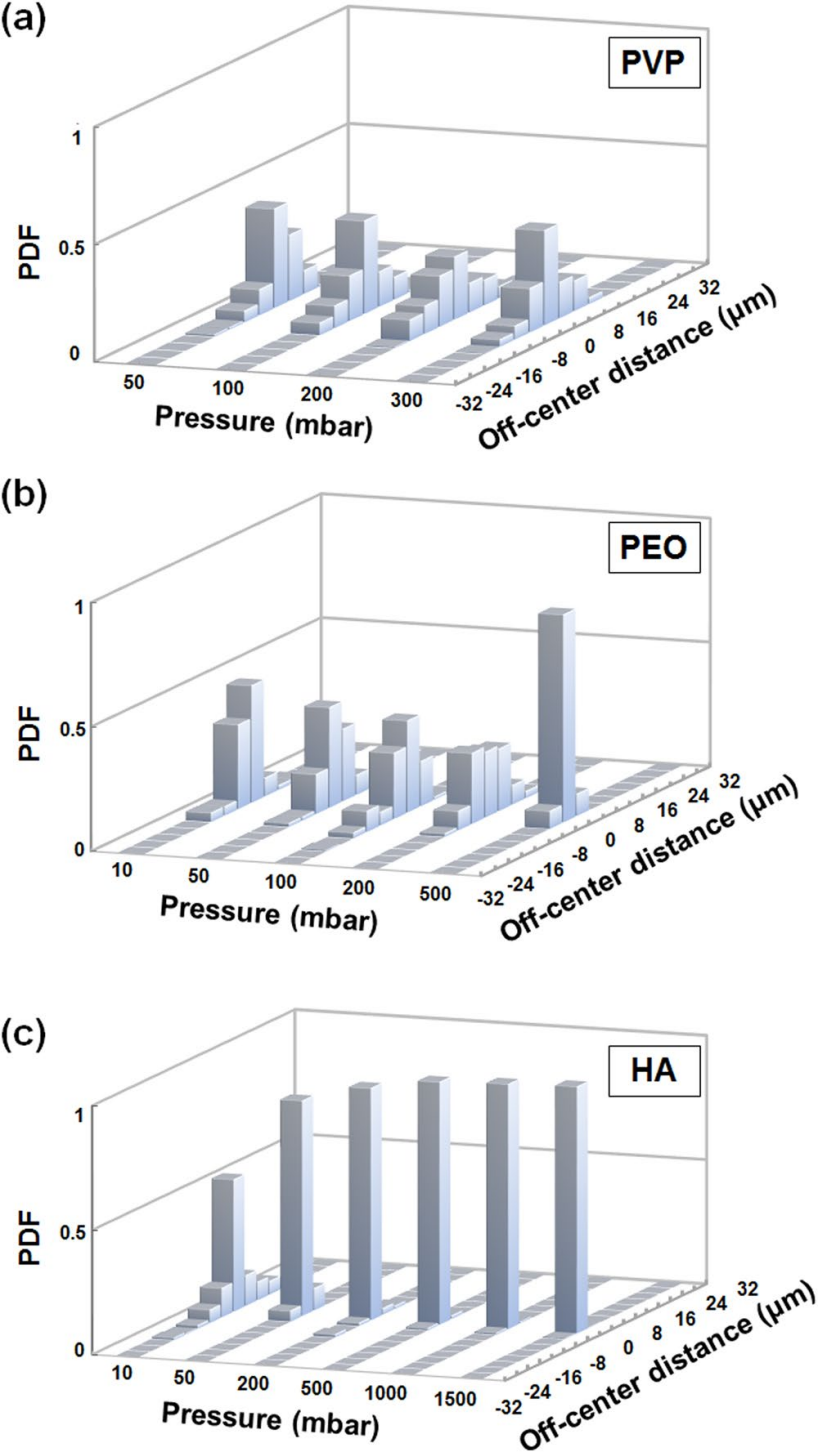
Probability distribution function (PDF) of particle distribution across the cylindrical capillary microchannel width (inner diameter = 60 µm) as a function of inlet pressure: (**a**) PVP, (**b**) PEO, and (**c**) HA solutions.

Figure 3a shows the focusing performance of PVP in the range of 0.039 < *Re* < 0.24, 0.00055 < *Wi* < 0.0034, and *El* = 0.014. Partial 3D focusing was observed with wide exponential tails in radial direction. Increasing the pressure does not provide any noticeable improvement, which can be explained with low elastic force on particles and thus low radial velocity. Figure 3b shows the PDF results for PEO in the range of 0.009 < *Re* < 0.45, 0.152 < *Wi* < 7.593, and *El* = 16.874. Successful 3D focusing was achieved when inlet pressure was increased from 200 mbar to 500 mbar. In Fig. 3c, PDF results for HA are given in the range of 0.00088 < *Re* < 0.2, 0.025 < *Wi* < 4.56, and 22.8 < *El* < 28.41. Here, we observed better 3D focusing performance compared to PEO and PVP even at low flow rates. Furthermore, the focusing efficiency is almost the same for inlet pressure values beyond 200 mbar. This provides a wide inlet pressure adjustment range, which is critical to obtain the highest throughput when designing the optical detection system.

### Experimental results on cytometry

**Figure 4:**
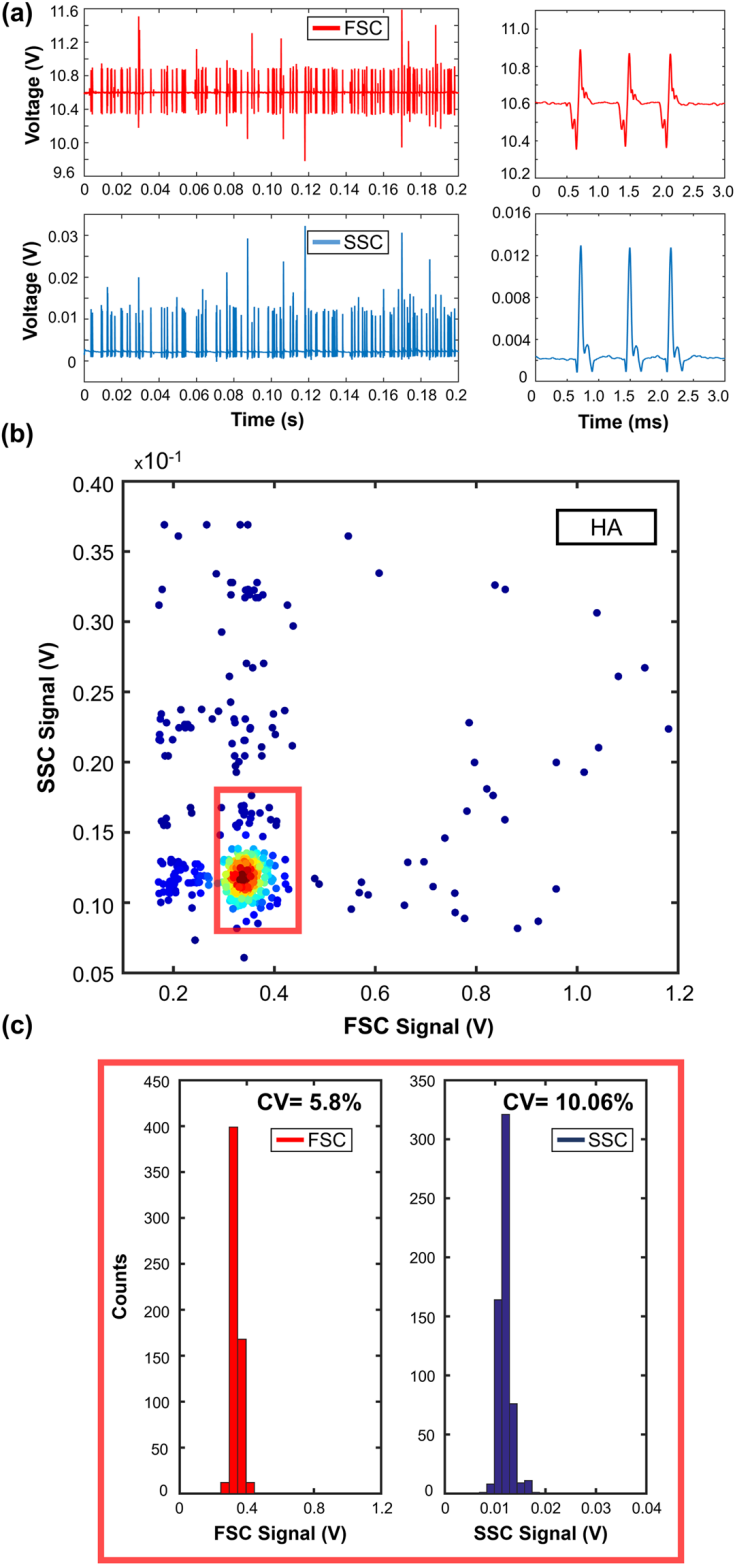
HA based viscoelastic flow cytometry results for 6 µm diameter polystyrene beads: (**a**) FSC and SSC signals together with 3 ms closer look-up. (**b**) Scatter plot of FSC vs. SSC events. Throughput is 750 events/s. 80% of the total events are populated inside the rectangular region. (**c**) Histograms of FSC and SSC signals; 5.8% CV and 10.06% CV obtained for FSC and SSC signals, respectively.

**Figure 5:**
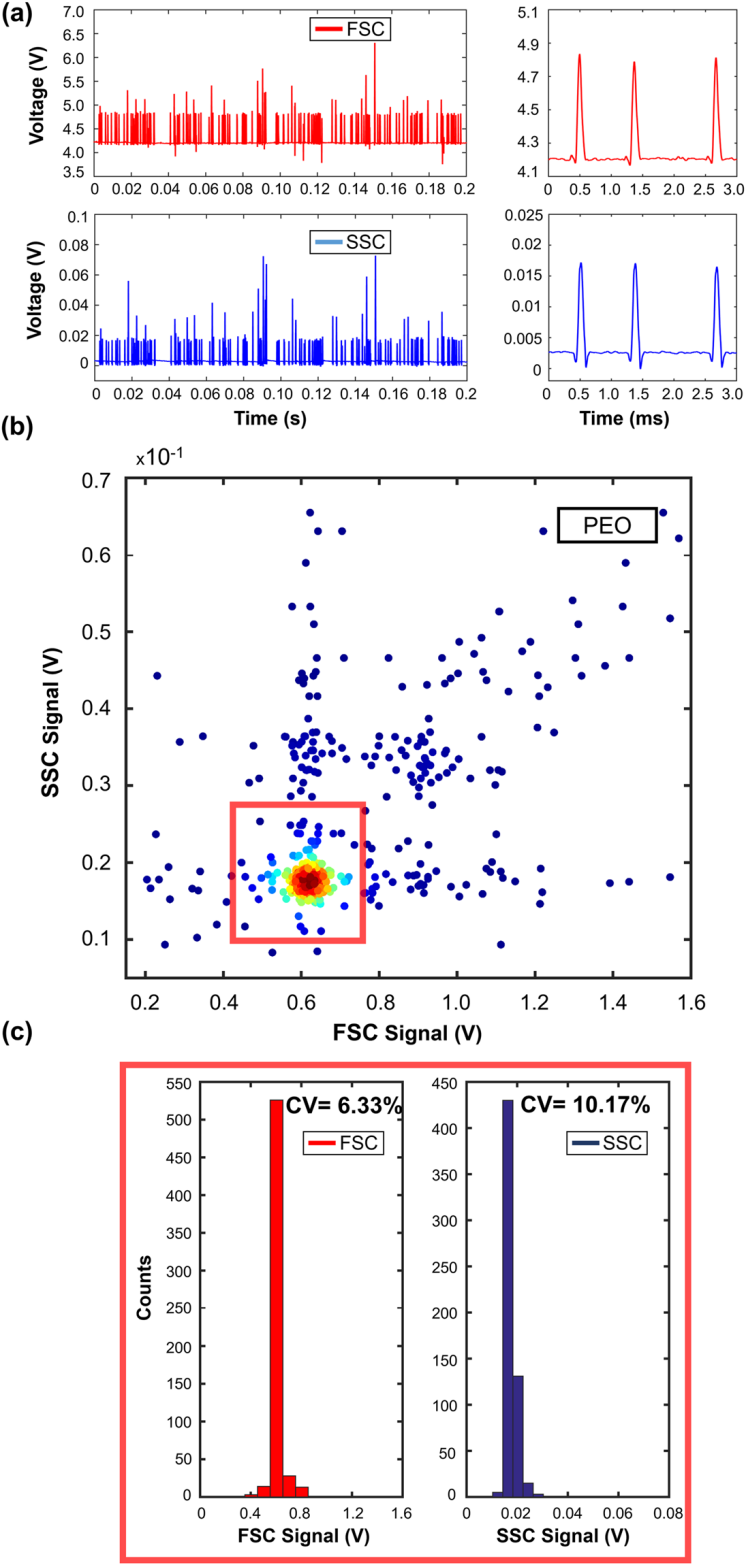
PEO based viscoelastic flow cytometry results: (**a**) FSC and SSC signals together with 3 ms closer look-up. (**b**) Scatter plot of FSC to SSC events. Throughput is 780 events/s. Above 80% of events populated in the rectangular region. (**c**) Histograms of FSC and SSC signals: 6.33% and 10.17% CV values were obtained for FSC and SSC signals, respectively.

**Figure 6:**
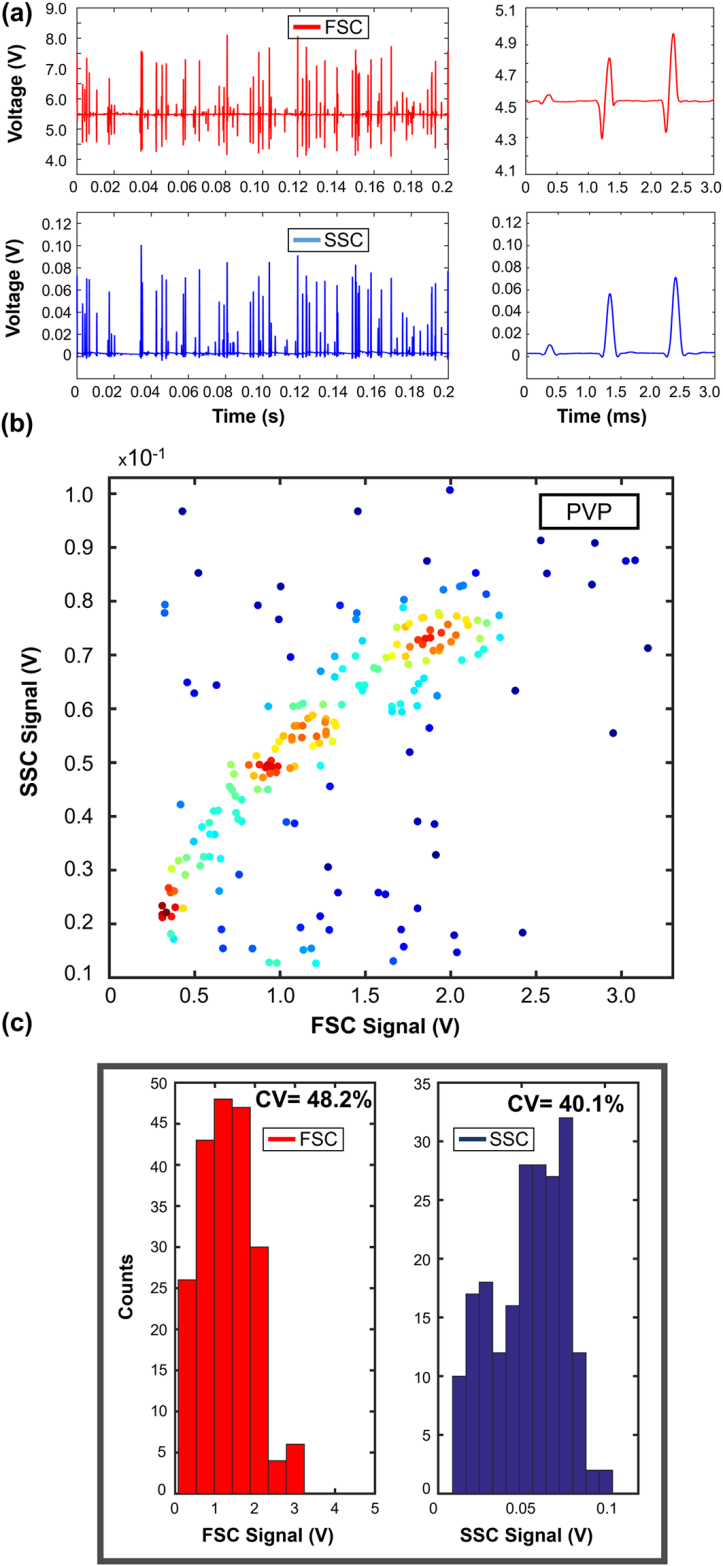
PVP based viscoelastic flow cytometry results: (**a**) FSC and SSC signals together with 3 ms closer look-up. (**b**) Scatter plot of FSC to SSC events. Throughput is 200 events/s. (**c**) Histograms of FSC and SSC signals without gating.

Both analytical calculations and experimental PDF values demonstrate that viscoelastic focusing is ideal for integrated sheathless microflow cytometers. Afterwards, we performed FSC and SSC signal measurements with the microflow cytometry device to compare the performance of three different viscoelastic fluids. The pressure values for HA, PEO, and PVP were set to 1000 mbar (Re = 0.11), 500 mbar (Re = 0.45), and 300 mbar (Re = 0.24), respectively. The distance between the illumination fiber and the capillary tube (30 µm) was adjusted to achieve 6 µm beam diameter to match the size of the particle that is focused at the center of capillary tube. Forward and side scatter light collecting fibers were 405 µm and 330 µm away from the capillary tube. The distance from capillary inlet to optical interrogation point was set as 6 cm, which is obtained from the experimental focusing measurements. The collected light was converted into voltage signal using two Si photodetectors. FSC signal collects some portion of the incident light, which leads to a non-zero base value for each sample. Figures 4–6 represent FSC and SSC measurement results obtained from HA, PEO and PVP solutions in descending cytometry performance. Data for 200 ms measurement duration are shown in Figs 4a, 5a and 6a together with the close-up views of three particle events in 3 ms measurement window. Scatter plots of FSC vs. SSC are given in Figs 4b, 5b, and 6b using FSC signal baseline reduction. Histograms together with the CV values are plotted in Figs 4c and 5c with rectangular gating, and Fig. 6c without gating for HA, PEO and PVP samples, respectively. The results of signal mean, standard deviation, and %CV are summarized in Table 2 for HA, PEO, and PVP based microflow cytometer.

**Table 2:** Flow cytometer results: mean, standard deviation, and coefficient of variation (%CV).

Solution	Mean (V)		Standard deviation (V)		% CV	
	FSC	SSC	FSC	SSC	FSC	SSC
HA	0.340	0.012	0.020	0.001	5.80	10.06
PEO	0.620	0.017	0.039	0.002	6.33	10.17
PVP	1.322	0.054	0.638	0.021	48.24	40.10

Figure 4a shows the signal using HA sample for both FSC and SSC, corresponding to 750 events/s of throughput. In closer look-up to an arbitrary 3 ms region, we observe that the particles are well separated and have 1 ms of periodicity. The FSC and SSC signals are accumulated at 10.9 V and 0.013 V, respectively. Only nine events in the entire 200 ms time duration had signal values greater than the voltage level of highly focused particles. These events include aggregates, doublets or triplets, yielding increase in measured signal. Figure 4b depicts scatter plot of events for 1 s time duration. It is evident that most of the counts are populated into a small region. To isolate doublets, triplets and clumps, a rectangular gating window was used, which removes the statistical outliers and includes more than 80% of the events. Figure 4c shows the histogram plots of the signal peaks with the gating, and the CV values were calculated as 5.8% and 10.06% for FSC and SSC, respectively.

Figure 5a represents the signals obtained using the PEO solution. The peaks are accumulated at 4.8 V for FSC and 0.016 V for SSC. Most of the counted events are populated into a small region; however, clumps are widely spread compared to HA solution as seen in Fig. 5b. The standard deviation, mean, and CV values of 6.33% and 10.17% intensity events were obtained for FSC and SSC, respectively. The throughput was obtained as 780 events/s.

For PVP solution, throughput was obtained as 200 events/s since we used a lower flow rate compared to other measurements. In Fig. 6b, FSC-SSC signal scatter plot has three accumulated regions. Weak 3D focusing performance of PVP causes fleeing particles, passing through the focused region without or weakly encountering with the illumination light. Detected beads show large signal variation and disrupted periodicity. There is no distinct boundary on counts for PVP sample. The low FSC signal region is due to fleeing particles and corresponds to most of the events. The rest of the events are widely spread in the scatter plot. Compared to the results obtained using PEO and HA solutions that are densely populated in the region of 0.3–0.7 V FSC and 0.01–0.02 V SSC, PVP events are very scarce in this region. Therefore, we conclude that the focusing of the particles is unsuccessful using PVP (M_w_ = 4 × 10^4^ Da).

Comparing the device performance and the resultant CV values, we see distinctively better performance with HA and PEO compared to PVP solution. Our analytical calculations, numerical simulations and focusing experiments are all in good agreement. In addition, our device shows close performance for both PEO and HA solutions with slightly higher CV values for HA. We also compared our HA microflow cytometry results with a commercial flow cytometer, BD Accuri C6. This system was calibrated before the experiment and gave a result of 3.5% CV for FSC signal and 13% CV for SSC signal with similar gating using 6 µm polystyrene particles suspended in DI water. Our HA based sheathless microflow cytometer gives 5.8% CV for FSC signal and 10.06% CV for SSC signal for the same particles. A detailed comparison between the two systems is given in Table 3. It can be seen that similar results are obtained from the two systems.

**Table 3:** Comparison of HA based sheathless microflow cytometer (HA-MFC) and commercial BD Accuri D6 flow cytometer for 6 μm particles.

	HA-MFC	BD Accuri D6
Throughput	~780 events/s	~240 events/s
Gating	80%	88%
CV for FSC	5.8%	3.5%
CV for SSC	10%	13%

**Figure 7:**
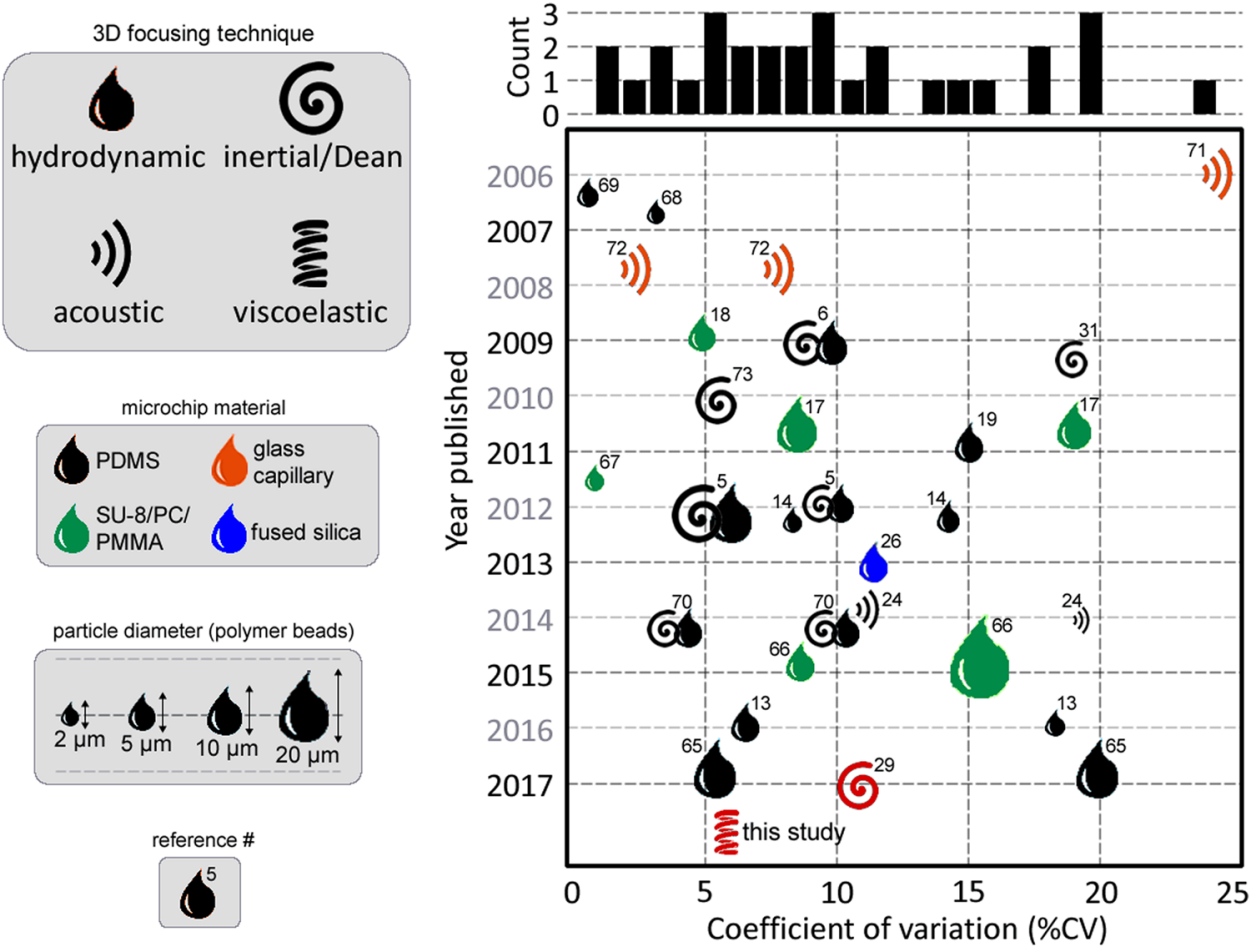
Comparison of the reported cytometers reported in the literature based on the focusing method (see Supplementary Table S4). The CV values reported for these values are from either forward scatter (FSC), side scatter (SSC) or fluorescence (FL) measurements (details are given in supplementary document). If multiple values were reported in the same study, the lower CV value (i.e. best performance) was reflected in the chart. For some studies, experiments were performed with differing size of particles, which is also reflected in the chart. The HA based sheathless microflow cytometer presented in this study yields a CV value of 5.8% for FSC measurement.

A figure of merit for comparison of flow cytometers is the CV values which are mostly obtained using polystyrene calibration microparticles. Despite the variation in the size of test particles or the measurement modes (forward scatter, side scatter or fluorescent detection), CV values provide a common ground for comparison of different systems. For the evaluation of our viscoelastic focusing based microflow cytometer, a comparison of the CV values reported for previously demonstrated microflow cytometers is given in Fig. 7. In general, three main focusing methods have been used for 3D focusing: hydrodynamic^8,13,14,17–19,26,65–69^, acoustic^24,71,72^, and inertial^28,29,43,73^ focusing. Some studies use a combination of these methods for focusing in two lateral dimensions^5,6,70^. For the studies listed in Fig. 7, we used the results obtained from polymeric test particles. We have plotted the best reported CV if multiple results are given during experimental optimization. A detailed list of these studies is given as supplementary information. As seen, there is a dispersion of CV values obtained using different focusing techniques. Most studies were able to obtain values below 10%. It is important to note that monodispersity of the test beads for these studies was not reported in most cases. Therefore, Fig. 7 should be used to overview the landscape of microflow cytometers rather than a strict comparison based on the CV values. Although this study presents the first attempt of combining viscoelastic focusing with microflow cytometry, we have achieved 5.8% CV. Considering the simplicity of the presented system, viscoelastic focusing is a promising approach for sheathless microflow cytometers. Such a system can also be equipped with high-end detection circuitries to match the throughput obtained using state-of-the-art commercial cytometers.

## Materials and Methods

### Device Design and Fabrication

Glass capillary tube with inner diameter (ID) of 60 µm and outer diameter (OD) of 190 µm was obtained from Polymicro Technologies. A single-mode optical fiber (core diameter 4.3 µm, cladding diameter 125 µm, S405-XP) was used as the delivery fiber, and two multi-mode optical fibers (core diameter ~62.5 µm, cladding diameter ~125 µm, M42L01) were used to collect optical signal. Since 125 µm OD capillary is not readily available off-the-shelf, we reduced the size of the 190 µm OD capillary tube to ~125 µm in order to match the level of the optical detection system to the center of the capillary tube (see Supplementary Fig. S1). To achieve fluidic access to the capillary, the capillary was inserted into a PDMS channel at the inlet. We carved guided grooves on a flat polymethyl methacrylate (PMMA) slab using CO_2_ laser (Epilog Zing, 30 W) for stabilizing the optical fibers and capillary microchannel. The microflow cytometer was finalized by simply assembling the capillary and the fibers together and fixing them onto the PMMA holder using adhesive tape (see Supplementary Fig. S2).

Figure 1 shows the schematic of all the components of the microflow cytometer. The input fiber was placed perpendicularly at one side of the capillary tube to guide the light through the capillary tube. On the opposite side, two detection fibers were placed at an angle of 13° and 30° to the incident light to collect the FSC and SSC signals, respectively. FSC fiber was oriented at a small angle to avoid the coupling of the direct beam from the light source^13^.

### Sample Preparation

**Figure 8:**
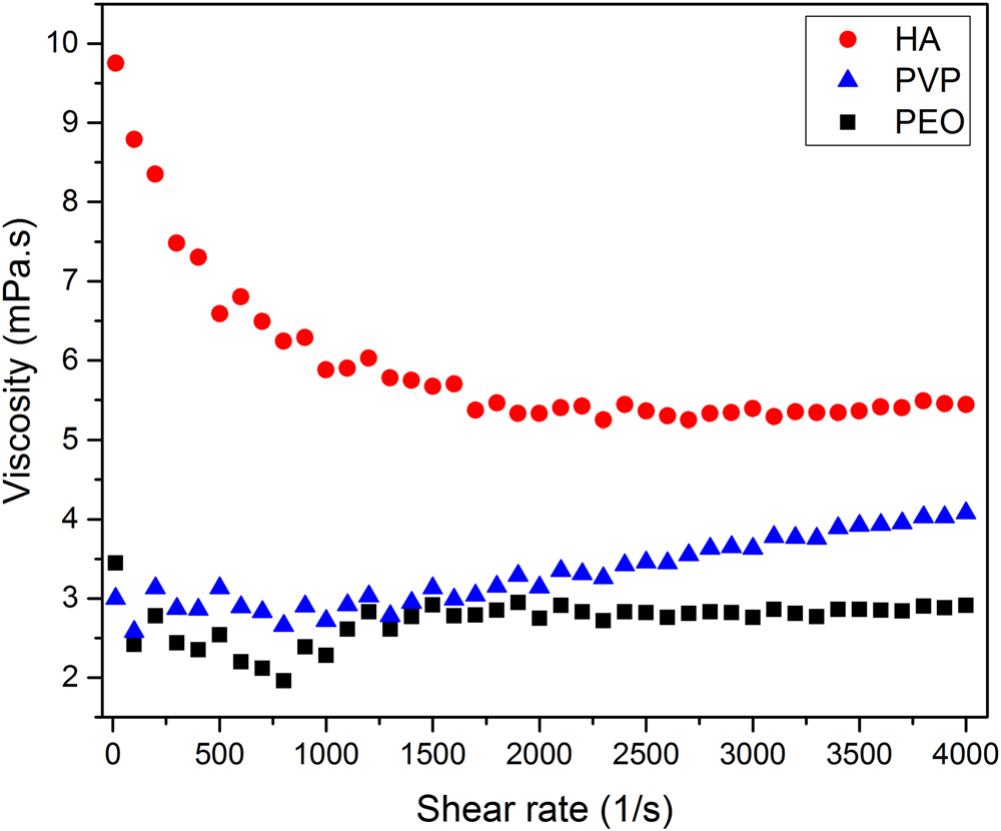
Viscosity of the viscoelastic fluids as a function of shear rate: 0.05% (w/v) PEO, 8% (w/v) PVP, and 0.5% (w/v) HA in water.

3D focusing using viscoelastic force is highly dependent on the rheological properties of the viscoelastic fluid. Therefore, it is critical to evaluate the performance of the microflow cytometer using different fluids. For this study, we have investigated three different biocompatible polymers to prepare viscoelastic fluids: polyethlyene oxide (PEO, M_w_ = 5 × 10^6^ Da, Sigma-Aldrich), polyvinylpyrrolidone (PVP, M_w_ = 4 × 10^4^ Da, Sigma-Aldrich) and hyaluronic acid (HA, M_w_ = 1.06 × 10^6^ Da, Binisu Schimoto). PEO, PVP, and HA were dissolved in DI water at 0.05% (w/v), 8% (w/v), and 0.5% (w/v) concentrations, respectively. We characterized the rheological properties of the viscoelastic fluids using a rheometer (Anton Paar, MCR 301). Figure 8 shows the viscosity of the fluids as a function of shear rate. PEO and PVP show nearly constant viscosity, especially in the shear rate range observed during our experiments ($$\dot{\gamma } < 2000\,{s}^{-1}$$ $$)$$. However, HA shows a shear thinning behavior. For the cytometry measurements, 6 µm diameter polystyrene microbeads (Polysciences, Inc.) were suspended in the viscoelastic solutions at a concentration of $$4\times {10}^{6}$$ particles/ml. The samples were continuously mixed to have uniform particle concentration throughout the experiments.

### Experimental Setup

We used a 635 nm laser source (Thorlabs, S1FC635) coupled to a single-mode input fiber. FSC and SSC collecting fibers were connected to two high-speed fiber coupled photodetectors (Thorlabs, DET02AFC). Voltage signals from photodetectors were measured using an oscilloscope (Techtronix, MDO3104) at 0.5 MHz sampling rate (Fig. 1a). The signal was post-processed with MATLAB to acquire peak values and apply gating if necessary. A pressure pump was used (Elveflow, OB1 2 bar) to deliver the polystyrene microsphere suspended viscoelastic fluids to capillary tube. The manipulation of the particles and their distribution inside the capillary tube were observed under an inverted microscope (Omano, OMFL600) attached to a high-speed camera (Vision Research, Phantom Miro M310). Particle position and distribution along the capillary tube were recorded with the high-speed camera and post-processing was done with ImageJ software.

### Numerical Simulation

We performed numerical simulations with COMSOL 5.2 to analyze the focusing effect of three viscoelastic fluids that have different relaxation times and viscosities. Since elastic force is mainly affected by ${N}_{1}$, ${N}_{2}$ is not considered in the simulations. Oldroyd-B model was used to define the stress due to the elastic effect. Navier-Stokes, continuity, and additional stress contribution in dimensionless form are defined as

8 $${\rm{Re}}(u.\nabla )u=\nabla .[-pI+{\mu }_{s}[\nabla u+(\nabla u){}^{T}]+T]$$

9 $$\nabla .(u)=0$$

10 $$T+Wi((u.\nabla )T-[(\nabla u)T+T{(\nabla u)}^{T}])={\mu }_{p}(\nabla u+{(\nabla u)}^{T})$$

where *Re*, *T*, ${\mu }_{s}$, ${\mu }_{p}$, and *u* correspond to Reynolds number, additional stress tensor, relative viscosity of solvent, relative viscosity of polymer and velocity, respectively. The relation between relative viscosities is

11 $${\mu }_{p}=\frac{{\eta }_{p}}{\eta }=1-{\mu }_{s}$$

Based on our results given in Fig. 8, relative solvent viscosities of PEO and PVP solutions (*µ*_*s*_) were taken as 0.357 and 0.333. On the other hand, HA behaves as a shear-thinning fluid, which is implemented using Carreau model as

12 $$\mu ={\mu }_{\inf }+({\mu }_{0}-{\mu }_{\inf }){[1+{(\lambda \dot{\gamma })}^{2}]}^{\frac{n-1}{2}}$$

The above equation was applied to our rheological measurement data shown in Fig. 8 for HA. Using curve fitting, the Carreau model variables were found as μ_*0*, *s*_ = 0.11, ${\mu }_{\inf ,{\rm{s}}}\,=\,0.2$, λ = 3.72 ms, and ${\rm{n}}=0$. By solving equations (8), (9) and (10) together, velocity field and normal stresses were obtained. Later, the first normal stress difference (${N}_{1}$) was found by subtracting ${\sigma }_{{rr}}$ from ${\sigma }_{{zz}}$ (equation (1)). Reynolds number was chosen as 0.01 to neglect inertia effect. Weissenberg numbers for PEO, PVP, and HA were calculated as 0.169, 0.000144, and 0.251, respectively. Using particle tracing module in COMSOL, radial position of the particles along the microchannel was obtained, which allowed us to study the focusing performance.

## Conclusion

In this study, viscoelastic focusing of microparticles for microflow cytometry is demonstrated. We devised a low-cost cytometer system composed of fibers and a straight capillary. Using the viscoelastic effect, we were able to achieve single-train focusing of microparticles. The performance of three viscoelastic solutions were analyzed using analytical, numerical and experimental methods. HA and PEO solutions perform very similar in achieving 3D focusing. Using numerical particle tracking, we were able to demonstrate the focusing results at different locations along the microcapillary. It was experimentally verified that HA_1.06MDa_ and PEO_5MDa_ solutions can focus 6 µm diameter particles in a single-line. We have also analyzed the elastic force analytically and quantified the viscoelastic effect using a rheological term that yielded very similar results to our numerical and experimental observations. We obtained FSC signal CV of 5.8% and 6.33% using HA and PEO, respectively. Thanks to its simplicity and performance, the presented system can have a wide range of uses in microflow cytometry applications.

## Electronic supplementary material

Supplementary Document

Supplementary Movie
