# Supplementary material for: Sheathless Microflow Cytometry Using Viscoelastic Fluids
Source: Sci Rep. 2017 Sep 27;7:12342. doi: 10.1038/s41598-017-12558-2 (PMC5617843; doi:10.1038/s41598-017-12558-2)
Supplement: Supplementary file 1 — Supplementary Document [file 41598_2017_12558_MOESM1_ESM.pdf]

SUPPLEMENTARY INFORMATION FOR
SHEATHLESS MICROFLOW CYTOMETRY USING VISCOELASTIC FLUIDS

Mohammad Asghari[†], Murat Serhatlioglu[†], Bülend Ortaç[†], Mehmet E. Solmaz[‡], and Caglar Elbuken [†]*

† UNAM - National Nanotechnology Research Center, Institute of Materials Science and Nanotechnology, Bilkent University, 06800, Ankara, Turkey.

‡ Department of Electrical and Electronics Engineering, Izmir Katip Celebi University, 35620, Izmir, Turkey

* Corresponding author: Caglar Elbuken

Tel: +90-312-2903550; Fax: +90-312-2664365; E-mail: elbuken@unam.bilkent.edu.tr

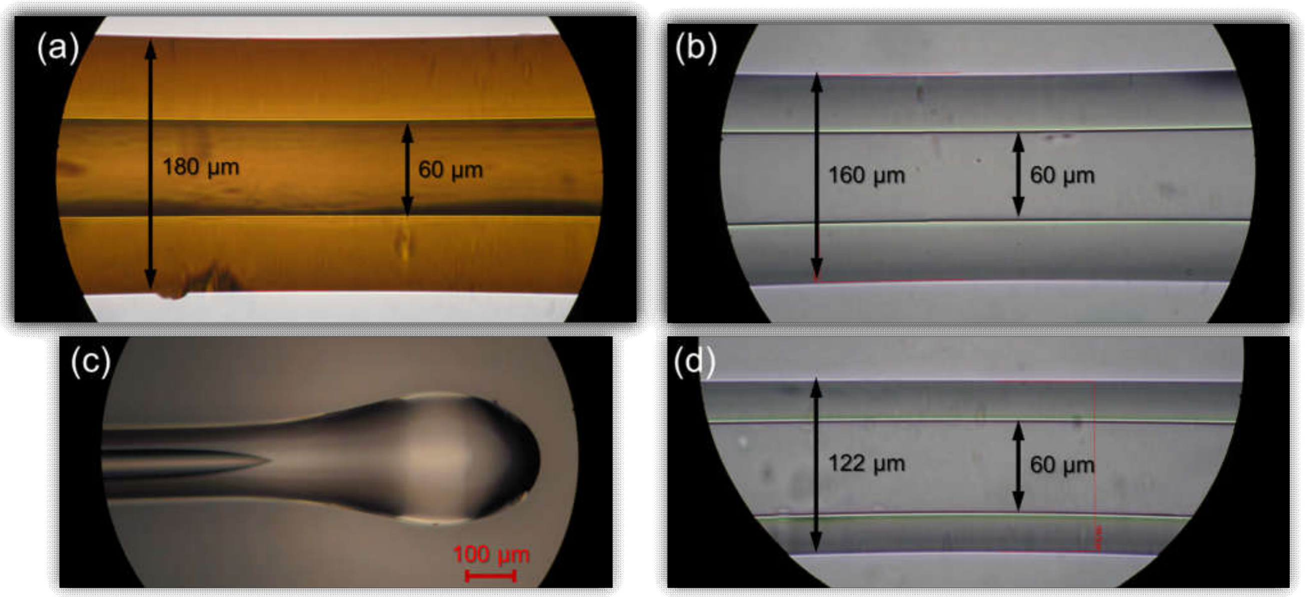

Figure S1. Preparation of capillary tube. (a) 160 μm OD, 60 μm ID capillary tube with 10 μm polyimide coating. (b) Polyamide coating was removed in sulfuric acid. (c) Ball lens protection cap to isolate the inner channel. (d) Capillary tube after 20 minutes of HF etching.

Alignment of capillary tube and fibers is crucial for flow cytometry measurement. We used a glass capillary tube (Figure S1a) with 160 μm outer diameter (OD) (after removing the ~10 μm polyimide coating) and optical fibers with 125±2 μm of OD in flow cytometer setup. The diameter of capillary tube was reduced to 122 μm in hydrofluoric acid solution (HF) to match the diameter of the optical fibers. First, 10 μm polyimide coating of capillary tube was stripped out in 95% sulfuric acid solution at 90 °C for 2 min (Figure S1b). Then, both ends of the capillary tube were melted to form a ball lens shape in a fusion splicer (Fujikara, FSM-100P) to protect the inside of the capillary during HF etching (Figure S1c). 15 cm long capillary tube was placed in 40% HF solution for 20 min and OD was reduced to 122 μm. Finally, ball lens ends of capillary tube were clipped. It is worth noting that capillary preparation step was required due to the unavailability of the desired capillary matching the OD of the available fibers.

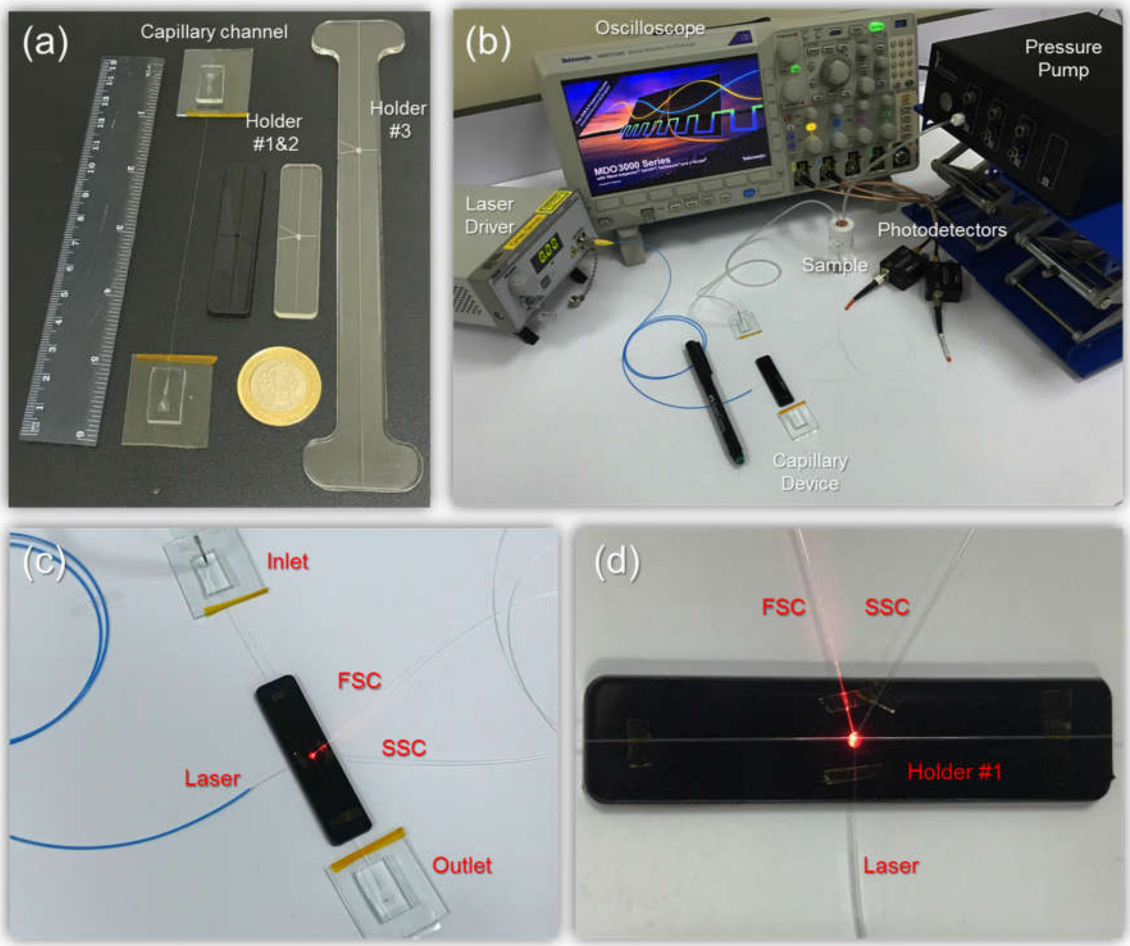

Figure S2. Viscoelastic microflow cytometry setup. (a) Capillary channel and PMMA holders (holder #1, #2 and #3) to assemble capillary tube and optical fibers. (b) Overview of experimental setup; capillary device, laser driver, oscilloscope, pressure pump, and photodetectors. (c) Closer look-up of viscoelastic microflow cytometer. (d) An image during the experiment (laser on) showing the optical interrogation region.

Table S1. Calculations of Re, Wi, and EI for PVP solution.

P (mbar)	P (Pa)	Q (m^3/s)	Q (µl/h)	Re	Wi	El
50	5000	5.30144E-12	19.08518	0.039375	0.000566	0.014381
100	10000	1.06029E-11	38.17035	0.07875	0.001133	0.014381
200	20000	2.12058E-11	76.3407	0.1575	0.002265	0.014381
300	30000	3.18086E-11	114.5111	0.23625	0.003398	0.014381

Table S2. Calculations of Re, Wi, and EI for PEO solution.

P (mbar)	P (Pa)	Q (m^3/s)	Q (µl/h)	Re	Wi	El
10	1000	1.13602E-12	4.08968	0.00904	0.152545	16.87407
50	5000	5.68011E-12	20.4484	0.045201	0.762723	16.87407
100	10000	1.13602E-11	40.8968	0.090402	1.525446	16.87407
200	20000	2.27204E-11	81.79361	0.180804	3.050893	16.87407
500	50000	5.68011E-11	204.484	0.452009	7.627232	16.87407

Table S3. Calculations of Re, Wi, and EI for HA solution.

P (mbar)	P (Pa)	Q (m^3/s)	Q (µl/h)	Re	Wi	El
10	1000	3.53429E-13	1.272345	0.000875	0.025333	28.95238
50	5000	1.76715E-12	6.361725	0.004922	0.126667	25.73545
200	20000	7.06858E-12	25.4469	0.020455	0.506667	24.77037
500	50000	1.76715E-11	63.61725	0.053938	1.266667	23.48360
1000	100000	3.53429E-11	127.2345	0.109375	2.533333	23.16190
1500	150000	5.30144E-11	190.8518	0.166373	3.8	22.84021
1800	180000	6.36173E-11	229.0221	0.202500	4.56	22.51852

Table S4. Comparison of the published microflow cytometers

Date	Authors	Reference no.	CV (%)	Focusing Method	Measurement mode *	Chip material **	Particle type ***	Particle diam. (µm)
Aug-17	Asghari et. al.	this work	5.8	viscoelastic	FSC	glass capillary	PS beads	6
Jan-17	Wang et. al.	29	11.2	inertial	FL	glass capillary	microbeads	10
Jan-17	Cheng et. al.	65	5.6	hydrodynamic	FL	PDMS	PS beads	15
Jan-17	Cheng et. al.	65	19.8	hydrodynamic	SSC	PDMS	PS beads	15
Oct-16	Zhao et. al.	13	6.3	hydrodynamic	FSC	PDMS	PS beads	10
Oct-16	Zhao et. al.	13	17.9	hydrodynamic	FSC	PDMS	PS beads	6
Dec-14	Testa et. al.	66	8.3	hydrodynamic	FL	PMMA	microbeads	10
Dec-14	Testa et. al.	66	15.4	hydrodynamic	FL	PMMA	microbeads	23
Jun-14	Nawaz et. al.	70	3.8	inertial & hydrodynamic	FSC	PDMS	PS beads	10
Jun-14	Nawaz et. al.	70	9.8	inertial & hydrodynamic	SSC	PDMS	PS beads	10
Jan-14	Chen et. al.	24	19.4	acoustic	FL	PDMS	PS beads	7.3
Jan-14	Chen et. al.	24	10.9	acoustic	FL	PDMS	PS beads	10
May-13	Erickson et. al.	26	11.6	hydrodynamic	FSC	fused silica	PS beads	10
Jul-12	Watts et. al.	14	8	hydrodynamic	FL	PDMS	PS beads	2.5
Jul-12	Watts et. al.	14	14	hydrodynamic	FL	PDMS	PS beads	6
Apr-12	Mao et. al.	5	6.3	inertial & hydrodynamic	FL	PDMS	PS beads	15.5
Apr-12	Mao et. al.	5	9.1	inertial & hydrodynamic	FSC	PDMS	PS beads	7.3
May-11	Frankowski et. al.	67	1.8	hydrodynamic	FL	PC	PS beads	3
Jan-11	Kennedy et. al.	19	15	hydrodynamic	FL	PDMS	PS beads	6
Jul-10	Rosenauer et. al.	17	8.4	hydrodynamic	TR	SU-8	PS beads	12
Jul-10	Rosenauer et. al.	17	19.1	hydrodynamic	TR	SU-8	PS beads	8
May-10	Oakey et. al.	73	5.6	inertial	FL	PDMS	PS beads	10.2
Nov-09	Bhagat et. al.	31	18	inertial	FL	PDMS	PS beads	6
Mar-09	Mao et. al.	6	9.3	inertial & hydrodynamic	FL	PDMS	PS beads	8.3
Jan-09	Kummrow et. al.	18	4.9	hydrodynamic	FL	PMMA/PC	PS beads	5
Nov-07	Goddard et. al.	72	2.5	acoustic	FL	glass capillary	PS beads	10
Nov-07	Goddard et. al.	72	7.7	acoustic	SSC	glass capillary	PS beads	10
Aug-06	Simonnet et. al.	68	3.4	hydrodynamic	FL	PDMS	PS beads	1.9
Jul-06	Bang et. al.	69	1.4	hydrodynamic	FL	PDMS	PU beads	3
Jan-06	Goddard et. al.	71	24	acoustic	FL	glass capillary	PS beads	10

* FSC = forward scatter SSC = side scatter FL = fluorescence TR = transmittance

** PDMS = polydimethyl siloxane PMMA = polymethyl methacrylate PC = polycarbonate

*** PS = polystyrene PU = polyurethane
